# Determination of THC and THC‐COOH in Dried Capillary Blood Spots and Comparison to Venous Blood of Recreational Cannabis Consumers in a Pilot Study

**DOI:** 10.1002/dta.70085

**Published:** 2026-05-12

**Authors:** Matthias Bantle, Willi Schirmer, Joel Bruegger, Thomas Wüthrich, Andreas Längin, Lena Zwicker, Reto Auer, Wolfgang Weinmann

**Affiliations:** ^1^ Institute of Forensic Medicine, Forensic Toxicology and Chemistry University of Bern Bern Switzerland; ^2^ Department of Chemistry, Biochemistry and Pharmaceutical Sciences University of Bern Bern Switzerland; ^3^ Graduate School of Cellular and Biomedical Sciences University of Bern Bern Switzerland; ^4^ Institute of Primary Health Care (BIHAM) University of Bern Bern Switzerland; ^5^ Center of Primary Care and Public Health (UNISANTÉ) Lausanne Switzerland

## Abstract

With increased sensitivity of instrumentation, more substances can be analysed using capillary dried blood spots (DBS), and therefore, minimal invasive sample collection can be performed. However, for cannabinoids such as (−)‐*trans*‐Δ^9^‐tetrahydrocannabinol (THC), low extraction efficiencies have been reported in previous studies. In this study, a novel extraction method was developed comprising an extraction with dimethyl sulfoxide followed by methanolic extraction, yielding extraction efficiencies > 80% for both THC and its metabolite 11‐nor‐9‐carboxy‐Δ^9^‐tetrahydrocannabinol (THC‐COOH). The linear ranges of the LC–MS/MS method are 0.5–20 ng/mL for THC and 1.25–100 ng/mL for THC‐COOH with good precision and accuracy. Concentrations of THC and THC‐COOH in DBS from capillary blood were compared to venous blood concentrations based on 159 blood samples retrieved from a study including recreational cannabis users. THC‐COOH concentrations in DBS were in close agreement with venous blood concentrations with a median capillary DBS/venous blood ratio of 1.09 (mean 1.10 ± 0.21), whilst THC concentrations in capillary blood were higher than venous blood concentrations with a median ratio of 2.11 (mean 16.6 ± 87.9) (after exclusion of outliers: 1.84 [mean 2.57 ± 1.93]), and the THC concentration ratio capillary DBS/venous blood showed a larger variability, ranging from 0.75 to 722 (0.75 to 8.18 after exclusion of outliers).

## Graphical abstract

A new extraction method was developed and validated for the extraction of THC and THC‐COOH from capillary blood samples collected as dried blood spots. The extracts were analysed using HPLC–MS/MS resulting in high extraction efficiencies for both THC and THC‐COOH.

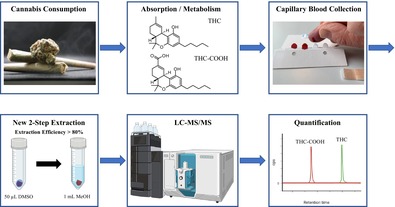

## 1 Introduction

Cannabis is among the most consumed recreational drugs worldwide. Its main psychoactive constituent is (−)‐*trans*‐Δ^9^‐tetrahydrocannabinol (THC). Despite its widespread consumption for recreational purpose, 244 million people between the age of 15 and 64 worldwide consumed cannabis in 2024 (4.6% of the global population aged between 15 and 64); it is prohibited in many countries [1]. In Switzerland, 7.6% of the population between the age of 15 and 64 consumed cannabis in 2022 [2]. As of 2021, there are pilot studies investigating the effect of controlled access to THC‐containing cannabis with a maximum THC content of 20% by dry weight in order to gain more scientific understanding regarding potential changes in the law [3, 4]. In the city of Bern, this study (SCRIPT Safer Cannabis—Research in Pharmacies randomized controlled trial, University of Bern, BASEC2022‐00733) started in 2024 with 700 participants [5].

THC and its major metabolites 11‐hydroxy‐Δ^9^‐tetrahydrocannabinol (11‐OH‐THC) and 11‐nor‐9‐carboxy‐Δ^9^‐tetrahydrocannabinol (THC‐COOH) can be detected in venous whole blood as well as serum [6, 7]. Recent developments for minimal invasive or on‐site sampling techniques use dried blood spots (DBS) from capillary blood as sample material. For alcohol biomarkers such as phosphatidylethanol (PEth), clinical parameters and many drugs of abuse, DBS analysis has successfully been implemented in the last years [8, 9, 10]. Some authors describe successful extraction of THC from DBS, whilst others found extraction efficiencies as low as 10%–20% [9, 11, 12, 13]. The aim was to develop a sensitive and robust extraction method for THC and its major inactive metabolite THC‐COOH from DBS and the application to real case samples of recreational consumers for comparison of capillary blood and venous blood concentrations.

## 2 Material and Methods

### 2.1 Participant Study

Detailed inclusion and exclusion criteria of SCRIPT participants are available elsewhere [14]. In short, participants had to be over 18 years of age, report regular cannabis use over the past six months, and, at the time of enrolment, their current cannabis use had to be confirmed by a urine test. Study nurses consented participants at the baseline visit in written form. The local ethics committee approved the trial and additional blood analyses (Decision number: 2022‐00733). At the study visit, which was independent from the last cannabis consumption, capillary blood (approx. 20 μL) from the fingertip was collected on Greencheck DBSV (Protzek, Germany) as well as venous blood using 7.5‐mL S‐Monovette EDTA Gel K2E (Sarstedt AG, Nümbrecht, Germany). The principle of the collection system and its comparability to classic DBS cards has been discussed by Stöth et al. [15]. The liquid blood samples were stored in aliquots of 500 μL in cryotubes (FluidX, Azenta Life Sciences, Burlington, NJ, USA) at −80°C until further analysis (later referred to as venous blood samples).

### 2.2 Analytical Part

All solvents were of HPLC‐quality. Dimethyl sulfoxide (DMSO, puriss. p.a., ≥ 99%) was obtained from Sigma‐Aldrich (Buchs, Switzerland). Methanol (MeOH, ≥ 99.9%) was obtained from Biosolve (Valkenswaard, Netherlands). Acetonitrile (MeCN, 99.9%) was obtained from Acros Organics (Geel, Belgium). Formic acid (50% in H_2_O) was purchased from Honeywell (Charlotte, NC, USA). Deionized water (18.2 MΩ·cm) was produced in‐house with a Milli‐Q system (Millipore, Billerica, MA, USA). External quality control samples were obtained from ACQ Science GmbH (Rottenburg a.N., Germany). Reference standards for (−)‐*trans*‐Δ^9^‐tetrahydrocannabinol (THC) and (±)‐11‐nor‐9‐carboxy‐Δ^9^‐*trans*‐tetrahydrocannabinol (THC‐COOH) were purchased from Merck Supelco (Darmstadt, Germany), and the internal reference standards (−)‐*trans*‐Δ^9^‐tetrahydrocannabinol‐D_3_ (THC‐D_3_) and (±)‐11‐nor‐9‐carboxy‐Δ^9^‐*trans*‐tetrahydrocannabinol‐D_3_ (THC‐COOH‐D_3_) were purchased from Lipomed (Arlesheim, Switzerland). Blank blood was obtained from the blood donation centre in Bern, Switzerland. External quality control samples were obtained from ACQ Science (Rottenburg a.N., Germany).

For calibration and for generation of an internal QC sample, standard solutions were spiked into blank blood for finally generating a seven‐point calibration ranging from 0.5 to 20 ng/mL for THC and an eight‐point calibration from 1.25 to 100 ng/mL for THC‐COOH, respectively. Aliquots of 20 μL of each calibration or quality control blood sample were applied to DBS cards (STERA, Basel, Switzerland) with a calibrated pipet (Gilson, Villiers Le Bel, France) and subsequently allowed to dry for at least 3 h. For the analysis of volunteers' capillary blood samples, one tooth of the DBSV card, with a capacity for 20 μL of blood, was detached and placed in a polypropylene (PP) vial (2 mL, Sarstedt, Nümbrecht, Germany). For the calibration and quality control (QC) samples, the whole spot was punched out using an in‐house built puncher with an internal diameter of 10 mm and then placed in a PP vial. Fifty microlitres of DMSO was added in each vial, and the vials were vortexed for 20 min (IKA Vibrax). Then, 1 mL of MeOH and 10 μL of deuterated standard solution containing THC‐D_3_ and THC‐COOH‐D_3_ were added, and the extraction mixture was shaken for 1 h, followed by centrifugation at 13,000 rpm and 8°C (Mikro 220, Hettich, Tuttlingen, Germany) for 10 min. The supernatant was then transferred to an auto sampler vial (Wicom, 2 mL, Heppenheim, Germany), and the MeOH phase was evaporated under a stream of N_2_ at 50°C for 45 min, with DMSO remaining in the vial. Then, 50‐μL reconstitution solvent (60% MeCN, 40% H_2_O, 0.1% formic acid) was added. The samples were analysed using LC–MS/MS consisting of a Nexera LC‐40B system (Shimadzu, Muttenz, Switzerland) coupled to a 7500 QTRAP (Sciex, Toronto, Canada) with an injection volume of 5 μL in positive mode using multiple reaction monitoring (MRM) at an ionisation voltage of 2200 V. The LC system consisted of two gradient systems, an extra pump for dilution of the injection volume and a two‐way‐six‐port valve for on‐line extraction. The method has been adapted from methods published by Hädener et al. and König et al. using a Kinetex C8 2.6 μm, 50 × 2.1 mm, 100 Å analytical column and a MercuryMS Synergi Polar RP 20 × 2 mm trapping column (Phenomenex, Torrance, CA, USA) [16, 17]. Gradient elution was performed using water with 0.1% formic acid (mobile phase A) and MeCN with 0.1% formic acid (mobile phase B) at an oven temperature of 40°C using the gradient scheme as depicted in Table S1. The MRM transitions and MS settings are described in Table S2. A two‐way‐six‐port switching valve installed in front of the MS‐source was used with the following timing: 0–3.5 min: LC ➔ waste, 3.5–6.5 min: LC ➔ MS, 6.5–12.0 min: LC ➔ waste. For the analysis of the venous blood samples, the method described by Hädener et al. and König et al. was used [16, 17].

### 2.3 Validation

Extraction efficiency was determined in triplicate from a comparison with pure reference solutions, and solutions spiked into blank blood and applied onto DBS cards at concentrations of 0.5, 1.5 and 20 ng/mL for THC and 2.5, 7.5 and 100 ng/mL for THC‐COOH. Carryover was determined by injecting a sample at a concentration of 20 ng/mL THC and 100 ng/mL THC‐COOH, respectively, followed by two injections of blank samples. Linearity and weighing (1/x) were determined by SciexOS (Version 3.4) in the calibration range. LOQ and LOD were determined applying a signal‐to‐noise (S/N) ratio of 10:1 for LOQ and 3:1 for LOD by extrapolation from calibrators at the LLOQ. Precision and accuracy have been determined from QC samples at 1.50 and 2.40 ng/mL for THC and 7.5 and 38.9 ng/mL for THC‐COOH by analysing 36 samples for each concentration. In addition, during the analysis series, the peak areas of deuterated internal standards have been monitored for testing of possible matrix effects.

## 3 Results

### 3.1 Participant Characteristics and Samples

Venous and DBS samples were collected from 90 and 159 participants, respectively, from the SCRIPT study. 89% (*N* = 142) reported using cannabis in the last 24 h.

### 3.2 Validation

**FIGURE 1:**
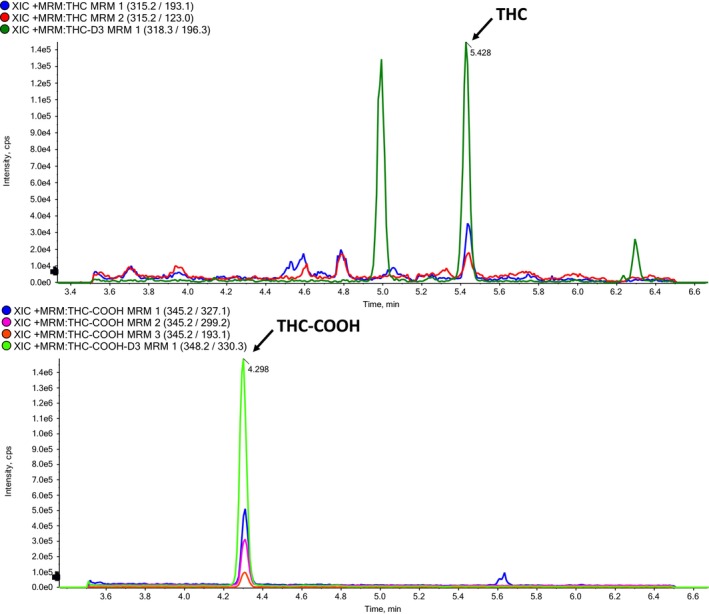
Extracted ion chromatogram (XIC) of THC (top) at 5.428 min (1.5 ng/mL) and for THC‐COOH (bottom) at 4.298 min (7.5 ng/mL).

**FIGURE 2:**
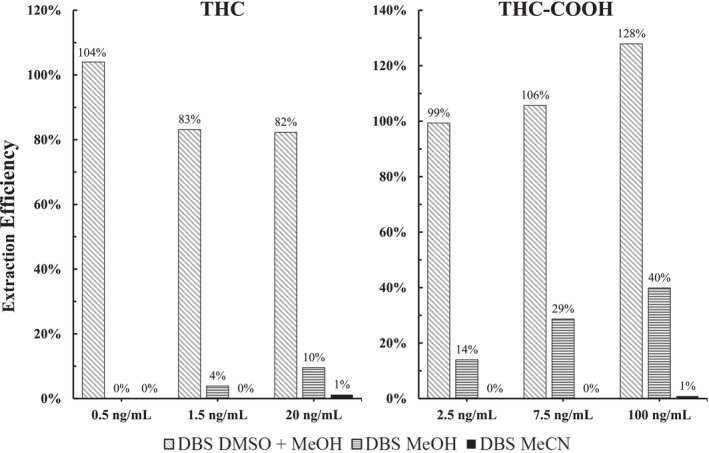
Comparison of extraction efficiencies of THC and THC‐COOH using DMSO + MeOH, MeOH, and MeCN. High extraction efficiencies greater than 80% and 99% for THC and THC‐COOH, respectively, were found by our new two‐step extraction with DMSO and MeOH. MeOH showed extraction efficiencies below 10% and 40% for THC and THC‐COOH, respectively. Extraction with MeCN yielded very low extraction efficiencies below 1.5% for both THC and THC‐COOH.

An LLOQ of 0.5 ng/mL for THC and 1.25 ng/mL for THC‐COOH was determined. The lowest calibration level for THC with 0.25 ng/mL was excluded, since the S/N ratio was below 10. In Figure 1, typical extracted LC‐MRM‐chromatograms are shown (1.5 ng/mL THC, 7.5 ng/mL THCOOH). Extraction efficiency was determined as 104% ± 6.5% (*N* = 3), 83% ± 2.7% (*N* = 3) and 82% ± 2.7% (*N* = 3) for THC at nominal concentrations of 0.5, 1.5 and 20 ng/mL, respectively, and 99.3% ± 22.1% (*N* = 3), 106% ± 11.6% (*N* = 3) and 128% ± 5.4% (*N* = 3) for THC‐COOH at nominal concentrations of 2.5, 7.5 and 100 ng/mL, respectively (Figure 2). In contrast, only low extraction efficiencies were found at the same concentrations if only MeOH or MeCN were used as extraction solvents (Figure 2).

For THC, precision and accuracy were determined as 12.2% (1.5 ng/mL), 13.6% (2.4 ng/mL), 1.6% (1.5 ng/mL) and 9.4% (2.4 ng/mL), respectively. For THC‐COOH, precision and accuracy were 7.2% (7.5 ng/mL), 8.3% (38.9 ng/mL), 6.3% (7.5 ng/mL) and 8.8% (38.9 ng/mL), respectively. Linearity was given for THC between 0.5 and 20 ng/mL and for THC‐COOH between 1.25 ng/mL and 100 ng/mL, respectively. Carryover has not been observed. For THC, an LOD of 0.25 ng/mL (S/*N* > 3:1) and LOQ of 0.5 ng/mL (S/*N* > 10:1) were determined. For THC‐COOH, an LOD of 0.5 ng/mL (S/N > 3:1) and an LOQ of 1.25 ng/mL (S/N > 10:1) were determined. The variations of signal intensities of the deuterated internal standards in an exemplary series of 54 authentic case samples were between 80 and 120% for both THC‐D_3_ and THC‐COOH‐D_3_. During method development, we tested the stability of THC‐COO‐glucuronide in DBS with reference standard spiked to blank blood at concentrations of 50 and 1000 ng/mL, respectively. No instability by cleavage of glucuronides has been observed directly after DBS preparation and after 10 days of storage of the DBS at −20°C.

### 3.3 THC and THC‐CCOH Concentrations in DBS and Venous Blood

**FIGURE 3:**
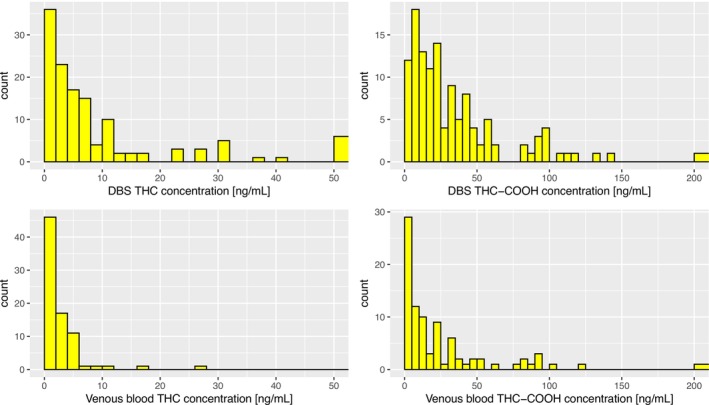
Histograms showing the concentration frequencies for THC (left, bin size = 2 ng/mL) and THC‐COOH (right, bin size = 5 ng/mL) for both DBS (top) and venous blood (bottom). Overflow bins are 50 ng/mL for THC (*n* = 6 for DBS, maximum concentration 490 ng/mL; *n* = 0 for venous blood) and 200 ng/mL for THC‐COOH (each *n* = 1 for DBS and venous blood, concentrations 288 and 283 ng/mL, respectively).

A total of 159 samples were analysed for THC and THC‐COOH on DBS. For THC, 111 (69.8%) were within the lower (0.5 ng/mL) and upper quantification (20 ng/mL) limits, 29 (18.2%) were below 0.5 ng/mL and 19 (11.9%) were exceeding 20 ng/mL. For THC‐COOH, 117 (73.6%) were within the lower (1.25 ng/mL) and upper quantification (100 ng/mL) limits, 36 (22.6%) were below 1.25 ng/mL and 6 (3.8%) were above 100 ng/mL. THC concentrations in DBS ranged from < LOD to 490 ng/mL (extrapolated) with a median of 4.32 ng/mL. THC‐COOH concentrations in DBS ranged from < LOD to 288 ng/mL (extrapolated) with a median of 23.3 ng/mL. In venous blood, concentrations ranged from < LOD to 26.7 ng/mL (extrapolated) (median = 0.80 ng/mL) for THC and from < LOD to 283 ng/mL (extrapolated) (median = 12.7 ng/mL) for THC‐COOH (Figure 3).

### 3.4 Ratio DBS/Venous Blood

**FIGURE 4:**
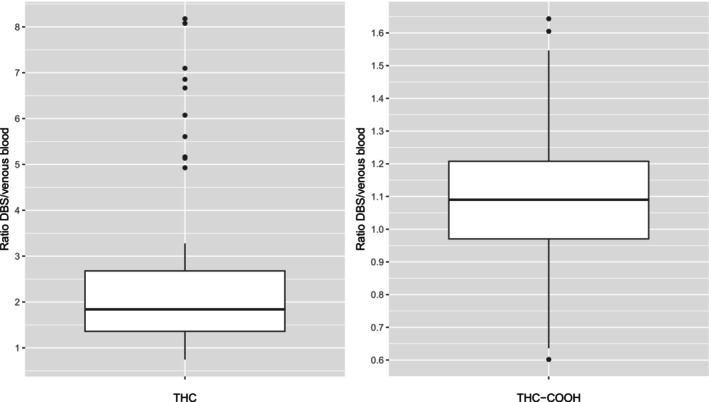
Ratios DBS/venous blood for THC (left) and THC‐COOH (right) after removal of outliers (*n* = 10 for THC, *n* = 0 for THC‐COOH).

From both venous blood and DBS, there were 67 and 66 quantitative results for THC and THC‐COOH, respectively. The concentration ratios DBS/venous blood for THC and THC‐COOH ranged from 0.75 to 722 and 0.60 to 1.64 with a median of 2.11 (mean 16.6 ± 87.9) and 1.09 (mean 1.10 ± 0.21), respectively. After removal of outliers (*n* = 10 for THC, *n* = 0 for THC‐COOH) according to Grubb's test (one‐sided, α = 0.05), the median concentration ratio DBS/venous blood for THC was 1.84 (mean 2.57 ± 1.93), ranging from 0.75 up to 8.18 (Figure 4).

## 4 Discussion

The enhanced extraction efficiency (> 80%) achieved by the two‐step extraction was found to be crucial for the sensitive determination of THC and THC‐COOH from capillary dried blood spots with an LLOQ of 0.5 ng/mL for THC and 1.25 ng/mL for THC‐COOH. In previously published methods, low extraction efficiencies of THC were found when only MeOH or MeCN were used as extraction solvents. However, Protti et al. and Mercolini et al. described extraction methods with extraction efficiencies > 80% for THC and THC‐COOH using MeOH only or in combination with MeCN, whilst Aydoğdu et al. only analysed the metabolite THC‐COOH [12, 13, 18]. Still, we could not reproduce these findings using only MeOH, as extraction efficiencies found for THC and THC‐COOH were below 10% and 40%, respectively (Figure 2). Low extraction efficiencies for THC were also described previously by Ververi et al. and by Thomas et al. [9, 11] In contrast to the methods published by Protti et al. and Mercolini et al. in our experiments, and in those of Ververi et al. and Thomas et al., the analytes were not applied onto spotted DBS from blank blood but spiked directly into blank blood before spotting onto filter paper cards and drying. This might explain the discrepancies between their findings (with extraction efficiencies of > 80%) and the low extraction efficiencies found by Ververi et al., Thomas et al. and by our own experiments (Figure 2). The sample preparation does not reflect reality if the analytes are added to blood after spotting onto filter paper. With the new two‐step extraction, DMSO has been used to dissolve the analytes from the dried blood spots on filter paper. Due to its amphipathic nature, DMSO is often used as solvent for hydrophobic molecules, and it has been used for cannabinoids in blood by GC–MS in combination with methylation [19, 20]. Furthermore, DMSO leads to swelling and dissolution of cellulose and therefore can enhance liberation of THC from the dried blood on filter paper [21]. Moreover, the addition of DMSO as an additive in liquid‐chromatography eluents may help to increase the ionisation efficiency of peptides with electrospray ionisation [22, 23, 24, 25]. The variation of internal standard signal intensity (80%–120%) indicates that no relevant matrix effects were present. However, they would be compensated using ion ratios of analyte/internal standard due to coelution and chemical similarity.

A large variability has been observed for the DBS/venous blood concentration ratio of THC, ranging from 0.75 to 722, whilst for the metabolite THC‐COOH, the ratio was in a narrow range with median and average in close agreement. For THC, 10 ratios have been classified as being outliers, using the alternative hypothesis that there are outliers amongst the highest values. A potential cause could be external contamination by THC on the fingertip of the participant arising from the consumption. A similar phenomenon has been described as a risk regarding the alcohol biomarker phosphatidylethanol (PEth): If ethanol was on the fingertip at the point of sample collection or if ethanol vapours (arising from disinfectants) were in the vicinity of the DBS sample during drying, post‐sampling formation of PEth has been observed [26]. However, in the case of cannabis, the possible contamination is not only THC from the plant material as such, but mainly its precursor, THC acid A, which is further decarboxylated to THC upon heating during consumption by smoking [27]. Still, due to the sensitive instrumentation and method with an actually quantified amount of THC in the range of picograms, even small quantities of THC on the skin of the fingertip could cause a measurable contamination. Furthermore, the used skin disinfection tissue contained glycerol and phenoxyethanol in water. Whilst this mixture is appropriate for puncture site disinfection, it is likely to be too polar to remove THC efficiently. Therefore, additional quantification of THC acid A would assist to confirm the cause of high DBS/venous blood concentration ratios. Alternatively, a different sampling location such as the earlobe could be used. The active metabolite 11‐OH‐THC, which is usually also quantified in venous blood in forensic cases, was not included in this method, since in preliminary experiments, we could not achieve enough sensitivity for this analyte in the lower concentration range.

## 5 Conclusion

In this study, a novel method was described to quantify both THC and its metabolite THC‐COOH from DBS with an extraction efficiency of 83% (median) for THC and a quantitative extraction (approx. 100%) for THC‐COOH with LLOQs of 0.5 and 1.25 ng/mL, respectively. Especially for THC, the extraction efficiency was significantly higher than with previously published methods, which is crucial for sensitive determination of THC in DBS. Regarding interpretation of results, care must be taken with respect to THC concentrations in DBS, as they might be subject to external contamination from the fingertip during sample collection. In future experiments, thorough decontamination of the puncture site must be performed prior to blood sampling, for example, by using other organic solvents (e.g., *iso*‐propanol) and additional detergents. Furthermore, THC acid A and its metabolites should be integrated in the LC–MS/MS method to distinguish between potential contamination and consumption. The metabolite THC‐COOH was not influenced by contamination problems and shows highly comparable results in venous blood and DBS from capillary blood.

## Funding

The blood was sampled among participants from the Safer Cannabis – Research In Pharmacies Randomized Controlled Trial (SCRIPT), supported by a grant (PLS 32003B_214832) from the Swiss National Science Foundation, a grant (TPF 326.5‐2/60) from the Swiss Tobacco Prevention Fund and the City of Bern. Internal funding at IRM, University of Bern, enabled analyses and interpretation of blood samples.

## Conflicts of Interest

The authors declare no conflicts of interest.

## Supporting information


**Table S1:** LC gradient settings. TC = trapping column, AC = analytical column, MS = mass spectrometer.
**Table S2:** MRM transitions and MS settings for THC and THC‐COOH in DBS. DP = declustering potential, EP = entrance potential, CE = collision energy, CXP = collision cell exit potential.

## Data Availability

The data that support the findings of this study are available from the corresponding author upon reasonable request.

## References

[1] United Nations Office on Drugs and Crime UNODC , “World Drug Report 2025,” accessed August 25, 2025, 2025, https://www.unodc.org/unodc/data‐and‐analysis/world‐drug‐report‐2025.html.

[2] Federal Office of Public Health , “Prevalence of Cannabis Consumption 2022,” 2024, https://ind.obsan.admin.ch/en/indicator/monam/cannabis‐consumption‐age‐15‐64.

[3] Federal Office of Public Health , “Pilot Trials With Cannabis,” 2025, https://www.bag.admin.ch/en/pilot‐trials‐with‐cannabis.

[4] Federal Government of Switzerland , “Verordnung über Pilotversuche nach dem Betäubungsmittelgesetz (BetmPV),” 2021, https://www.fedlex.admin.ch/eli/cc/2021/217/de.

[5] Federal Office of Public Health , “SCRIPT – Cities of Bern, Biel, Lucerne,” 2024, https://www.bag.admin.ch/en/script‐cities‐of‐bern‐biel‐lucerne.

[6] M. A. Huestis , J. E. Henningfield , and E. J. Cone , “Blood Cannabinoids. I. Absorption of THC and Formation of 11‐OH‐THC and THCCOOH During and After Smoking Marijuana,” Journal of Analytical Toxicology 16, no. 5 (1992): 276–282, 10.1093/jat/16.5.276. 1338215

[7] P. Kelly and R. T. Jones , “Metabolism of Tetrahydrocannabinol in Frequent and Infrequent Marijuana Users,” Journal of Analytical Toxicology 16, no. 4 (1992): 228–235, 10.1093/jat/16.4.228. 1323733

[8] K. A. Walker , T. K. Rudd , J. N. Vignola , et al., “Evaluation of Dried Blood Spot Sampling for Verification of Exposure to Chemical Threat Agents,” Forensic Toxicology 43, no. 2 (2025): 280–293, 10.1007/s11419-025-00721-8. 40232631 PMC12241275

[9] C. Ververi , C. Gentile , M. Massano , A. Salomone , and M. Vincenti , “Quantitative Determination by UHPLC‐MS/MS of 18 Common Drugs of Abuse and Metabolites, Including THC and OH‐THC, in Volumetric Dried Blood Spots: A Sustainable Method With Minimally Invasive Sampling,” Journal of Chromatography B 1247 (2024): 124337, 10.1016/j.jchromb.2024.124337. 39401474

[10] S. Mestria , S. Odoardi , V. Valentini , et al., “Metabolism Study of 3‐Chloromethcathinone (3‐CMC) by Dried Blood Spot (DBS) Sampling After Controlled Administration Using a Murine Model,” Drug Testing and Analysis 17, no. 6 (2025): 772–778, 10.1002/dta.3782. 39081107 PMC12151706

[11] A. Thomas , H. Geyer , W. Schänzer , et al., “Sensitive Determination of Prohibited Drugs in Dried Blood Spots (DBS) for Doping Controls by Means of a Benchtop Quadrupole/Orbitrap Mass Spectrometer,” Analytical and Bioanalytical Chemistry 403, no. 5 (2012): 1279–1289, 10.1007/s00216-011-5655-2. 22231507

[12] M. Protti , J. Rudge , A. E. Sberna , G. Gerra , and L. Mercolini , “Dried Haematic Microsamples and LC–MS/MS for the Analysis of Natural and Synthetic Cannabinoids,” Journal of Chromatography B 1044‐1045 (2017): 77–86, 10.1016/j.jchromb.2016.12.038. 28088044

[13] L. Mercolini , R. Mandrioli , V. Sorella , et al., “Dried Blood Spots: Liquid Chromatography–Mass Spectrometry Analysis of Δ9‐Tetrahydrocannabinol and Its Main Metabolites,” Journal of Chromatography A 1271, no. 1 (2013): 33–40, 10.1016/j.chroma.2012.11.030. 23228918

[14] University of Bern , “The Safer Cannabis—Research In Pharmacies Randomized Controlled Trial (SCRIPT),” 2023, https://clinicaltrials.gov/study/NCT06120855.

[15] F. Stöth , K. Koch , M. Bantle , P. Pütz , F. Wortmann , and W. Weinmann , “Increase of PEth After Single Consumption of Alcohol and Evaluation of a Volumetric DBS Filter Paper Device,” Journal of Analytical Toxicology 47, no. 4 (2023): 379–384, 10.1093/jat/bkad009. 36790103

[16] M. Hädener , W. Weinmann , S. Schürch , and S. König , “Development of a Rapid Column‐Switching LC‐MS/MS Method for the Quantification of THCCOOH and THCCOOH‐Glucuronide in Whole Blood for Assessing Cannabis Consumption Frequency,” Analytical and Bioanalytical Chemistry 408, no. 7 (2016): 1953–1962, 10.1007/s00216-016-9311-8. 26781107

[17] S. König , B. Aebi , S. Lanz , M. Gasser , and W. Weinmann , “On‐Line SPE LC‐MS/MS for the Quantification of Δ9‐Tetrahydrocannabinol (THC) and Its Two Major Metabolites in Human Peripheral Blood by Liquid Chromatography Tandem Mass Spectrometry,” Analytical and Bioanalytical Chemistry 400, no. 1 (2011): 9–16, 10.1007/s00216-011-4708-x. 21311875

[18] M. Aydoğdu , H. Ertaş , F. N. Ertaş , and S. A. Akgür , “Liquid–Liquid Extraction Solvent Selection for Comparing Illegal Drugs in Whole Blood and Dried Blood Spot With LC–MS–MS,” Journal of Analytical Toxicology 49, no. 1 (2024): 26–35, 10.1093/jat/bkae081. PMC11753396 39366924

[19] J. Galvao , B. Davis , M. Tilley , E. Normando , M. R. Duchen , and M. F. Cordeiro , “Unexpected Low‐Dose Toxicity of the Universal Solvent DMSO,” FASEB Journal 28, no. 3 (2014): 1317–1330, 10.1096/fj.13-235440. 24327606

[20] P. Kintz and V. Cirimele , “Testing Human Blood for Cannabis by GC–MS,” Biomedical Chromatography 11, no. 6 (1997): 371–373, 10.1002/(SICI)1099-0801(199711)11:6<371::AID-BMC685>3.0.CO;2-Y. 9413618

[21] A. Koura , H. Schleicher , and B. Philipp , “Untersuchungen Zur Quellung und Lösung von Cellulose in Aminhaltigen Flüssigkeitsgemischen: 2. Mitt.: Untersuchungen Zur Löslichkeit von Cellulose in Dimethylsulfoxid/Amin‐Gemischen,” in Band 23, Heft 3 März 1972, eds. C. Erich , et al. (De Gruyter, 1972), 128–133, 10.1515/9783112517949-006.

[22] W. Chang , S. Yan , X. Yan , et al., “The Sensitive Detection of Low Molecular Mass Peptide Drugs in Dried Blood Spots by Solid‐Phase Extraction and LC‐HRMS,” Analytical and Bioanalytical Chemistry 416, no. 26 (2024): 5655–5669, 10.1007/s00216-024-05480-w. 39180594

[23] H. Hahne , F. Pachl , B. Ruprecht , et al., “DMSO Enhances Electrospray Response, Boosting Sensitivity of Proteomic Experiments,” Nature Methods 10, no. 10 (2013): 989–991, 10.1038/nmeth.2610. 23975139

[24] C. Görgens , S. Guddat , A. Thomas , and M. Thevis , “Recent Improvements in Sports Drug Testing Concerning the Initial Testing for Peptidic Drugs (< 2 kDa)—Sample Preparation, Mass Spectrometric Detection, and Data Review,” Drug Testing and Analysis 10, no. 11–12 (2018): 1755–1760, 10.1002/dta.2503. 30239151

[25] J. G. Meyer and E. A. Komives , “Charge State Coalescence During Electrospray Ionization Improves Peptide Identification by Tandem Mass Spectrometry,” Journal of the American Society for Mass Spectrometry 23, no. 8 (2012): 1390–1399, 10.1007/s13361-012-0404-0. 22610994 PMC6345509

[26] A. Bashilov , S. Osipenko , K. Ikonnikova , et al., “False Positive Results of Phosphatidylethanol (PEth) Quantitation in Dried Blood Spots (DBS): The Influence of Alcohol Vapors,” Separations 9, no. 9 (2022): 250, 10.3390/separations9090250.

[27] J. Jung , M. R. Meyer , H. H. Maurer , C. Neusüß , W. Weinmann , and V. Auwärter , “Studies on the Metabolism of the Δ9‐Tetrahydrocannabinol Precursor Δ9‐Tetrahydrocannabinolic Acid A (Δ9‐THCA‐A) in Rat Using LC‐MS/MS, LC‐QTOF MS and GC‐MS Techniques,” Journal of Mass Spectrometry 44, no. 10 (2009): 1423–1433, 10.1002/jms.1624. 19728318

