# Supplementary material for: Determination of THC and THC‐COOH in Dried Capillary Blood Spots and Comparison to Venous Blood of Recreational Cannabis Consumers in a Pilot Study
Source: Drug Test Anal. 2026 May 12;18(7):905–11. doi: 10.1002/dta.70085 (PMC13327133; doi:10.1002/dta.70085)
Supplement: Supplementary file 1 — Table S1: LC gradient settings. TC = trapping column, AC = analytical column, MS = mass spectrometer. Table S2: MRM transitions and MS settings for THC and THC‐COOH in DBS. DP = declustering potential, EP = entrance potential, CE = collision energy, CXP = collision cell exit potential. [file DTA-18-905-s001.docx]

**Supplementary materials**

Table S1: LC gradient settings. TC = trapping column, AC = analytical column, MS = mass spectrometer

| **Time (min)** | **Pumps 1 and 2: analytical column (AC)** | | **Pumps 3 and 4, Pump 5: trapping column (TC)** | | | **Switching valve** |
| --- | --- | --- | --- | --- | --- | --- |
|  | Mobile Phase B (%) | Flow rate (µL/min) | Mobile Phase B (%) | Flow rate (µL/min) | Flow rate Pump 5 (µL/min) |  |
| 0.00 | 30 | 300 | 50 | 500 | 300 | TC🡪waste  AC🡪MS (load) |
| 1.00 |  |  |  |  |  | TC🡪AC🡪MS (elute) |
| 1.20 | ↓ (linear gradient) |  |  |  |  |  |
| 1.30 |  |  |  | 20 | 20 |  |
| 7.50 | 97.5 |  |  |  |  | TC🡪waste, AS🡪MS (re-equilibration) |
| 7.60 |  |  | 100 | 1000 |  |  |
| 10.00 |  |  |  |  |  |  |
| 10.10 | 30 |  | 50 | 500 | 300 |  |
| 12.00 |  |  |  |  |  | TC🡪waste  AC🡪MS |

Table S2: MRM transitions and MS settings for THC and THC-COOH in DBS. DP=Declustering potential, EP=entrance potential, CE=collision energy, CXP=collision cell exit potential.

| **Analyte** | **Q1 / Da** | **Q3 / Da** | **DP / V** | **EP / V** | **CE / V** | **CXP / V** |
| --- | --- | --- | --- | --- | --- | --- |
| THC MRM1 | 315.200 | 193.100 | 30 | 10 | 30 | 15 |
| THC MRM2 | 315.200 | 123.000 | 30 | 10 | 42 | 15 |
| THC-COOH MRM1 | 345.200 | 327.143 | 30 | 10 | 21 | 22 |
| THC-COOH MRM2 | 345.200 | 299.156 | 30 | 10 | 26 | 24 |
| THC-COOH MRM3 | 345.200 | 193.126 | 30 | 10 | 36 | 13 |
| THC-D_3_ MRM1 | 318.280 | 196.300 | 30 | 10 | 29 | 4 |
| THC-COOH-D_3_ MRM1 | 348.240 | 330.300 | 30 | 10 | 19 | 4 |
